# Application of quinoline derivatives in third-generation photovoltaics

**DOI:** 10.1007/s10854-021-06225-6

**Published:** 2021-07-09

**Authors:** Gabriela Lewinska, Jerzy Sanetra, Konstanty W. Marszalek

**Affiliations:** 1 Institute of Electronics, Faculty of Computer Science, Electronics and Telecommunications, AGH University of Science and Technology, 30-059, Kraków, Poland; 2 The author Jerzy Sanetra is retired from Institute of Physics, Faculty of Materials Science and Physics, Cracow University of Technology, 30-035, Kraków, Poland

## Abstract

Among many chemical compounds synthesized for third-generation photovoltaic applications, quinoline derivatives have recently gained popularity. This work reviews the latest developments in the quinoline derivatives (metal complexes) for applications in the photovoltaic cells. Their properties for photovoltaic applications are detailed: absorption spectra, energy levels, and other achievements presented by the authors. We have also outlined various methods for testing the compounds for application. Finally, we present the implementation of quinoline derivatives in photovoltaic cells. Their architecture and design are described, and also, the performance for polymer solar cells and dye-synthesized solar cells was highlighted. We have described their performance and characteristics. We have also pointed out other, non-photovoltaic applications for quinoline derivatives. It has been demonstrated and described that quinoline derivatives are good materials for the emission layer of organic light-emitting diodes (OLEDs) and are also used in transistors. The compounds are also being considered as materials for biomedical applications.

## Introduction

Due to increased energy demand, new methods of energy production are being explored with the ultimate goal of developing emission-free energy. Fossil fuels detrimentally affect the natural environment and introduce about 20 × 10^12^ kg of carbon dioxide into the atmosphere [1, 2]. The world is increasing, often turning, towards renewable energy [3–5], and more specifically towards solar radiation [6–10]. At present, silicon-based photovoltaic panels are the most commonly used [11–13]. Although the production of these cells is easy to manage, it requires a relatively high temperature which in turn affects the final costs. An alternative to inorganic solar cell materials are organic polymer compounds [14–17]. While their optoelectronic properties are the same as those of conventional semiconductors, they have excellent mechanical properties and plastic characteristics (e.g. ease of production, shape, malleability, colouring, etc.) [18–22]. For photovoltaic applications, materials with specific optoelectronic [23, 24] and mechanical [25] properties are needed.

**Fig. 1:**
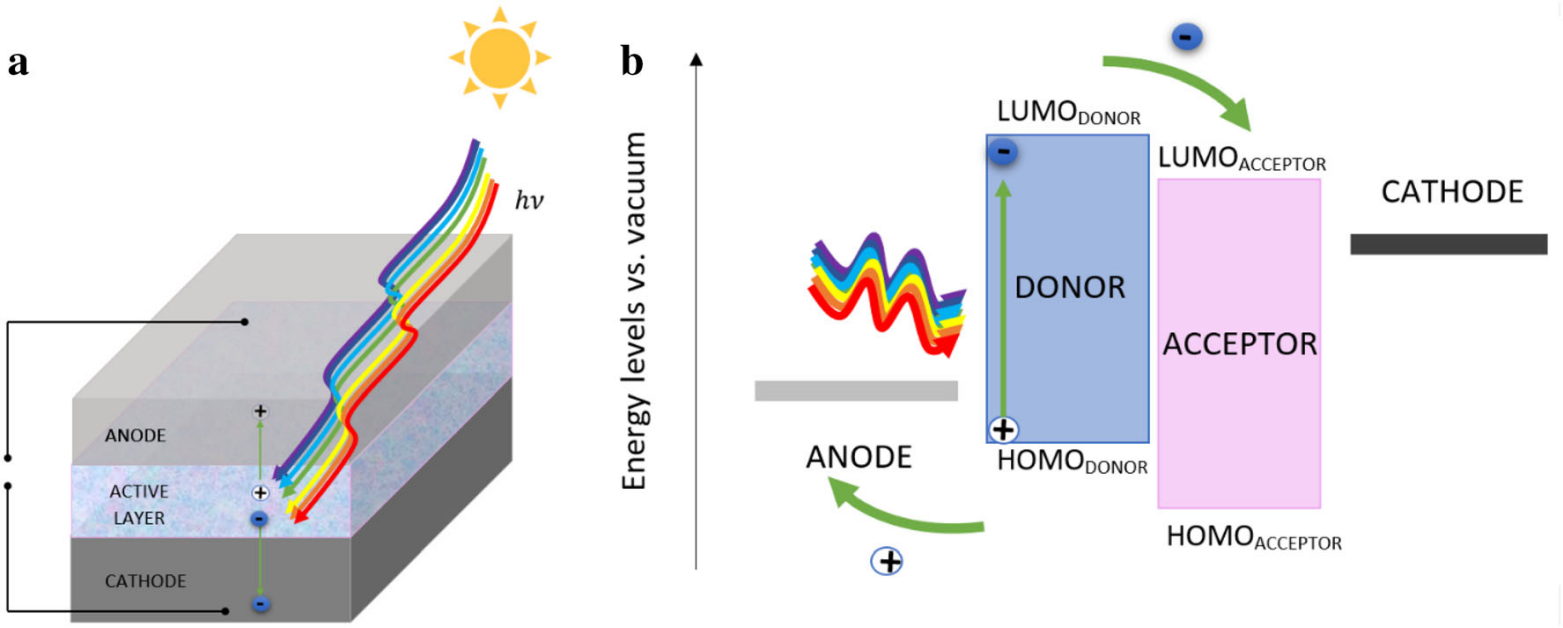
Heterojunction polymer solar cell; **a** architecture, and **b** energy diagram

One dynamically developing area of photovoltaics is polymer photovoltaics. Among the compounds being considered in this space are quinoline derivatives, which lend themselves to the modifications needed to optimize their properties. Typical materials in this class are thiophene [26, 27], fullerene derivatives [28, 29], benzothiadiazole-based polymers [30, 31], rylenediimide-based polymers [32, 33], and poly(phenylenevinylene) polymers [34, 35]. However, materials with new properties not displayed by the existing compounds are still needed and sought after. The quinoline derivatives are promising for applications in polymer photovoltaic solar cells and dye-synthesized solar cells (DSSCs). We divide the cells into polymer solar cells, oligomer solar cells, and small molecule solar cells. A schematic architecture of the organic cell and the principle of operation (charge transport following the energy levels) is shown in Fig. 1. The material most often used to make transparent electrodes is indium tin oxide (ITO) [36, 37], but fluorine tin oxide (FTO) is also used [38–42]. These materials are frequently treated and modified [43–48]. Other common electrode materials are tungsten oxide [49–51] or materials based on graphene [52, 53] and nanotubes [54–56]. The second electrode is usually made of metal [57–59]. To improve the efficiency of the cells, some additional materials are used between the electrodes to block the electrons [60–62] or to block the holes [63–66]. The function of the material, however, is not predefined; for example, supporting materials can be used as electrodes. One such material is poly(3,4-ethylenedioxythiophene) polystyrene sulfonate (PEDOT:PSS), which is used both as a support layer [67, 68] and as an electrode material [69]. When the same material is used for different functions, protective layers and encapsulation techniques are used to preserve the cells [70–73].

**Fig. 2:**
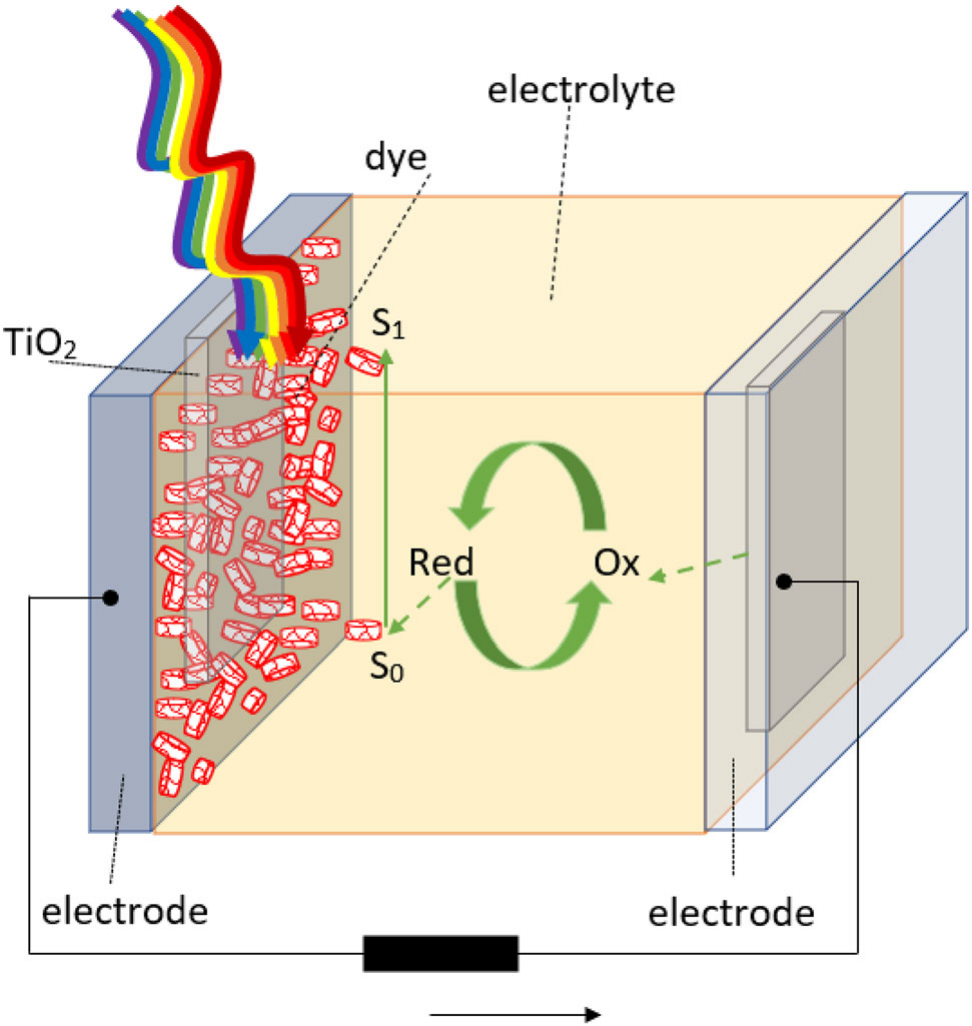
The architecture of DSSCs

DSSCs are another type of photovoltaic cells in which quinoline derivatives are used. A mechanism similar to that underlying DSSCs function is observed in plants, i.e. photosynthesis, which is the production of organic compounds in cells containing chlorophyll or bacteriochlorophyll. DSSCs are constructed of photo-anodes (dye-sensitized metal), redox vapour (e.g. I^−^/triodide /I^3−)^ dissolved in a suitable medium (electrolyte), and cathodes (which must be made of a material that can catalyse the redox reaction). Both electrodes can be transparent or semitransparent, allowing the solar cell to be lit from many directions. Usually, a platinum layer is used as a cathode deposited on a substrate (FTO). Carbon materials (graphene nanotubes) are also often used. Figure 2 presents the working scheme of DSSCs.

As with all photovoltaic processes, a cell’s performance begins with light absorption, in this case by dye particles, which causes electrons to move from their basic states to the excited states. Then, the electrons are injected into regeneration by oxidation I^−^ to I^3−^). In the last stage, the charge is transported in a porous layer of the semiconductor to the conductive electrode, and then via an external circuit to the cathode [75].

Research on photovoltaic cells is focused on new manufacturing methods, alternative architectures, and investigation of new materials for both the electrode and active layer components. Quinoline derivatives are one of the materials of interest. They are easily soluble, chemically stable, and miscible in organic solvents. The synthesis process has already been mastered. Therefore, a great interest in quinoline derivatives has been observed among scientists and for this reason this overview of quinoline derivatives used in 3rd generation photovoltaics, selected from recent years, is dedicated to them.

## Materials and properties

**Fig. 3:**
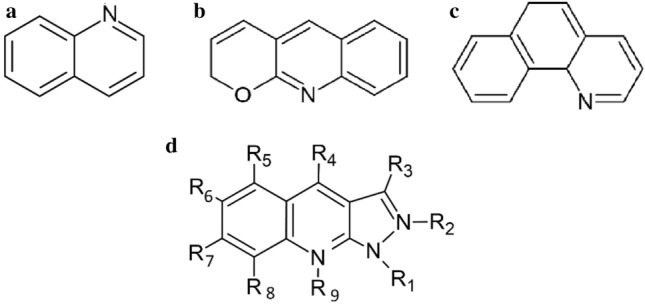
Chemical structure: **a** quinoline; **b** 2H-pyrano[2,3-b] quinoline; **c** benzo[h] quinoline, structure adapted from[30]; **d** 1H-pyrazolo[3,4-b]quinoline derivatives (R is substituent); structure adapted from [78]

Quinoline is a photoactive liquid which browns under the influence of light [74]. It is used as a solvent, antiseptic, flavouring, a dye, and even a preservative [75]. Chemically, quinoline is a heterocyclic aromatic organic compound (C_9_H_7_N). Its structure is based on a heterocyclic ring containing ten delocalized π electrons. Although the quinoline compound itself is well studied, its derivatives undergo continuous development (see examples in Fig. 3). The control over the quinoline derivatives synthesis process allows researchers to achieve specific optoelectronic properties of these compounds [76]. For example, researchers who wish to improve light harvesting, which requires extending the absorption spectrum to longer wavelengths, use electron-deficient units, such as pyridine, pyrimidine, benzotriazole, thiazole, or quinoxaline moieties [77]. Quinoline-based cell solutions achieving increasingly better performance II are an ongoing theme.

**Fig. 4:**
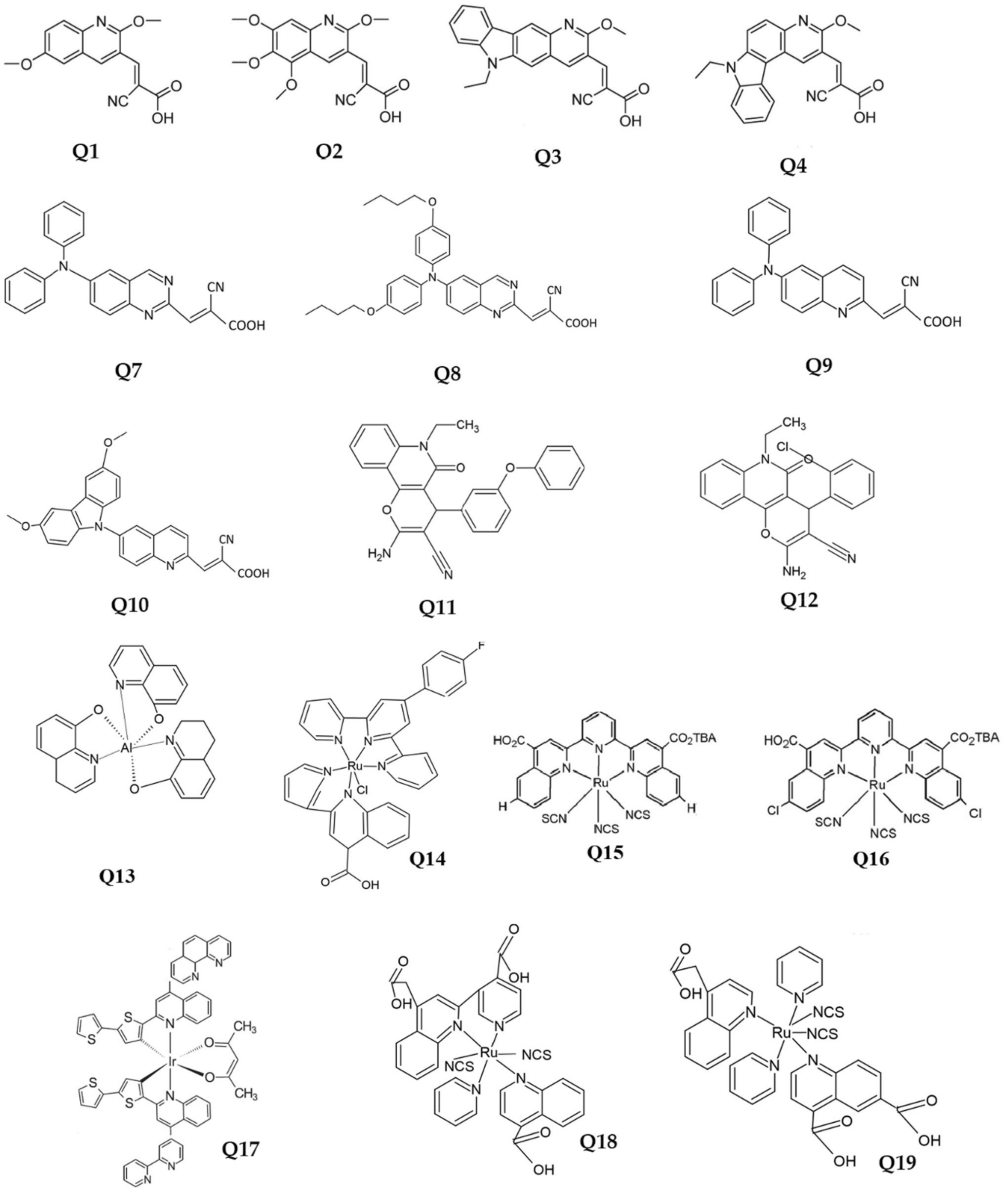
Quinoline derivatives and their chemical formulas: organic compounds (2-cyano-3-(2,6-dimethoxyquinolin-3-yl) acrylic acid (Q1), 2-cyano-3-(2,5,6,7-tetramethoxyquinolin-3-yl)acrylic acid (Q2), 2-cyano-3-(6-ethyl-2-methoxy-6H-pyrido[3,2-b]car- bazol-3-yl)acrylic acid (Q3), 2-cyano-3-(7-ethyl-3-methoxy-7H-pyrido[2,3-c]carbazol-2-yl)acrylic acid (Q4), 2-methyl-N,N-diphenylquinazolin-6-amine (Q5),N,N-bis(4-butoxyphenyl)-2-methylquinazolin-6-amine (Q6),2-methyl-N,N-diphenylquinolin-6-amine (Q7),*N*,*N*-bis(4-butoxyphenyl)-2-methylquinolin-6-amine (Q8),(E)-2-cyano-3-(6-(diethylamino)quinolin-2-yl)acrylic acid (Q9),(E)-2-cyano-3-(6-(3,6-dimethoxy-9H-carbazol-9-yl)quinolin-2-) acrylic acid (Q10), 2-amino-6-ethyl-5-oxo-4-(3-Ph)-5, 6-dihydro-4H-pyrano[3,2-c]quinoline-3-carbonitrile (Q11), 2-amino-4-(2-Cl)-6-ethyl-5-oxo-5,6-dihydro-4H-pyrano[3,2-c]quinoline-3-carbonitrile (Q12) and metal complexes (tris-8-hydroxy-quino- linato aluminium (Alq3) (Q13),[Ru(p-F-tpy)(pcqH)Cl]PF6 (F-TPY 40-(4-fluoro phenyl)- terpyridine, pcqH 2- (2-pyridyl)-4-carboxyquinoline) (Q14), 2,6-bis(4-carboxy-5-chloroquinolin-2-yl)pyridine (Q15), [Ru{2,6-bis(4-carboxy-5-chloroquinolin-2-yl) pyridine}Cl3] (Q16), 2 - bis[2-(2,2′-bithien-5-yl)-4-phenylquinolinato-C4,N]iridium(III) (2,4-pentanedionato-O2,O4) - [Ir(q-bt-Ph)2(acac)] (Q17),4-carboxy-2-(2′-pyrdyl) quinoline (Hmcpq) (Q18), cis-[Ru(H2dcpq)2(NCS)2] (2;H2dcpq′4-carboxy-2-[2′-(4′-carboxypyridyl)]quinoline) (Q19))

Figure 4 shows the latest quinoline derivatives designed for photovoltaic applications, due to its chemical structure. The organic compounds are tagged Q1–Q12 and the metal complexes are marked Q13–Q19. Arslan et al. [78] synthesized four novel dyes (designated Q1–Q4). These structures contain a quinoline moiety as a π-spacer, and cyanoacrylic acid. Their synthesis was motivated by the idea that the delocalization degree of the dye can be increased by extending π-spacers from quinoline to pyridocarbazole. Mao et al. presented a series of organic dyes (designated Q5, Q6) based on quinoline as an electron-deficient π-linker, and introduce push–pull conjugated dyes, synthesized by Riley oxidation of –CH_3_ followed by Knoevenagel condensation [79]. The four dyes bearing either quinazoline or quinoline as conjugated bridges in the chromophore with a diphenylamine moiety were synthesized (designated Q7–Q10 [80]).

Two interesting derivates—2-amino-6-ethyl-5-oxo-4-(3-Ph)-5,6-dihydro-4H-pyrano[3,2-c]quinoline-3-carbonitrile (Q11) and 2-amino-4-(2-Cl)-6-ethyl-5-oxo-5,6-dihydro-4H-pyrano[3,2-c]quinoline-3-carbonitrile (Q12)––have been presented by Zeyda et al. [81], who tested material properties and manufactured photovoltaic solar cells.

Kao et al. [82] reported organic solar cells based on tris-8-hydroxy-quino-linato aluminium (Alq3) designated as Q13, a fluorescent dye that is an excellent organic electroluminescent material, in which Alq3 was used as a dopant. Tolkki et al. [83]. Also demonstrated applications of tris(8-hydroxyquinoline)aluminium complexes as buffer layers in photovoltaic solar cells. Mongal et al. [84] described the properties of chemical compounds tested experimentally and density functional theory (DFT) simulation results. The authors give an overview of the properties and applications of terpyridine- and pyridine-quinoline-based mixed chelate ruthenium dye (Q14). Onozawa-Komatsuzaki et al. [85] reported a new ruthenium(II)–polypyridyl complex with a 2,6-bis(4-carboxyquinolin-2-yl)pyridine ligand, which is delocalized and electronegative, thereby lowering the energy level and extending the absorption spectrum. In recent years, star-shaped molecular materials have also been described. 2-bis[2-(2,2′-bithien-5-yl)-4-phenylquinolinato-C4,N]iridium(III) (2,4-pentanedionato-O2,O4) (Q17) was synthesized and applied in photovoltaic polymer cell by Szafraniec-Gorol et al. [86]. Ruthenium complexes (Q18, Q19) have been synthesized for the first time, and their spectra and electrochemical properties were obtained by Yanagida et al. [87].

Polymer solar cells are constructed of nanometer metallic and transparent electrodes and the so-called active layer [88]. Quinoline derivatives are most commonly used as materials in the active layer. The materials—both the donor and the acceptor—must fulfil a number of requirements, for which they need to be extensively tested [89].

The first stage of the photovoltaic process is absorption. UV–Vis spectroscopy is used to measure the absorption spectrum of chemical compounds as well as to examine the emission spectra, most often the fluorescence spectrum [90–92]. Photofluorescence indicates that a compound can create a metastable excited state, with a lifetime of about 10^−8^ s. The shift of the maximum absorption band relative to the emission band for the same excited state is called the Stokes shift [93].

It is, therefore, important to determine the Highest Occupied Molecular Orbital (HOMO) and Lowest Unoccupied Molecular Orbital (LUMO) levels for newly synthesized pyrazoloquinoline derivatives. These levels are a critical factor in the transport of charges generated by the photovoltaic process. Cyclic voltammetry is defined as the dependence of the current on the potential when a linearly changing potential is applied to the operating electrode [94]. Another popular method of determining HOMO and LUMO levels is the combination of ultraviolet photoelectron spectroscopy (UPS) and X-ray photoelectron spectroscopy (XPS), thus, UPS/XPS [95, 96]. This method measures the kinetic energy of the photoelectrons emitted by molecules as a result of their absorption of ultraviolet photons. In the process, the valence electrons are knocked out. The detector registers the photoelectrons emitted by the monochromatic X-ray source and the ultraviolet source. HOMO–LUMO transition occurs in the valence band region, so the excitation photon energy of a few volts is required. To determine the mobility of the charges in the junction, impedance spectroscopy [97] or Hall effect measurements [98] are usually carried out. These properties can also be obtained using calculation methods (DFT and different variants of DFT) [99].

Besides absorption in the active layer, reflection and transmission of the light beam are also possible [100]. In the tests of electrodes and supporting layers, a transparent electrode should have the highest transmission coefficient. It is also important to trap the photon in the active layer, so the active layer—the adjacent layer—must be properly chosen for the reflection coefficient [101]. These rates may also be tested with a spectrophotometer.

Geometrical parameters of a photovoltaic cell play a key role in its operation. To optimize the cell’s performance, the thickness of the layers must be adjusted properly. Due to the cell manufacturing procedure (layer-by-layer application), it is important for both wet and vacuum techniques to control the roughness of each obtained layer. The roughness influences the reflection factor (dispersion) as well as the process of applying the next layer. These parameters can be controlled with atomic force microscopy measurements (AFM) [102, 103]. Because of its nondestructive character, spectroscopic ellipsometry (SE) [104] is a useful research technique. SE is used to obtain the dispersion dependencies of the refractive and extinction coefficients, layer thicknesses, and structure of multilayer structures.

For photovoltaic applications, possible compounds are tested for their thermal stability. The compounds are required to present no temperature-generated molecular disorders in the range of − 20 °C to approximately 80 °C [105, 106]. Phase transitions are thus tested using differential scanning calorimetry (DSC), and the degradation temperature is determined using thermogravimetry.

Tables 1 and 2 specify the absorption bands, HOMO and LUMO levels, and possible additional properties presented in publications concerning selected quinoline derivatives. Most pyrazoloquinoline derivatives have absorption bands ranging from UV to red light. However, the spectrum of the derivative described by Onozawa-Komatsuzaki et al. [85] and Yanagida et al. [87] is wide—up to 900 nm, covering almost the entire solar spectrum. The magnitude of the energy gap correlates with the absorption spectrum. Although the utility of energy levels cannot be determined without the context of the cell architecture, the presented energy levels are within the requirements of solar cell applications.

**Table 1:** Quinoline derivatives (organic compounds) and their properties

Chemical compound	Reference	Absorption bands [nm]	Energy levels		Others
			LUMO [eV]	HOMO [eV]	
2-cyano-3-(2,6-dimethoxyquinolin-3-yl) acrylic acid	[78]	350–600	1.10	− 2.24**	DFT calculations, photon-to-current conversion efficiency
2-cyano-3-(2,5,6,7-tetramethoxyquinolin-3-yl) acrylic acid	[78]	350–600	1.07	− 1.80**	
2-cyano-3-(6-ethyl-2-methoxy-6H-pyrido[3,2-b]car- bazol-3-yl)acrylic acid	[78]	350–610	1.02	− 1.79 **	
2-cyano-3-(7-ethyl-3-methoxy-7H-pyrido[2,3-c]carbazol-2-yl)acrylic acid	[78]	350–620	1.00	− 1.46**	
2-methyl-N,N-diphenylquinazolin-6-amine	[79]	370–570	1.06	− 1.25**	
N,N-bis(4-butoxyphenyl)-2-methylquinazolin-6-amine	[79]	370–600	1.18	− 1.00**	
2-methyl-N,N-diphenylquinolin-6-amine	[80]	370–600	1.13	− 1.20**	
N,N-bis(4-butoxyphenyl)-2-methylquinolin-6-amine	[80]	370–600	1.19	− 1.00**	
(E)-2-cyano-3-(6-(diethylamino)quinolin-2-yl)acrylic acid	[80]	280–600	1.28	− 1.29**	Electrochemical impedance spectroscopy (EIS) charge recombination process
(E)-2-cyano-3-(6-(3,6-dimethoxy-9H-carbazol-9-yl)quinolin-2-) acrylic acid	[80]	280–590	1.20	− 1.28**	
2-amino-6-ethyl-5-oxo-4-(3-Ph)-5, 6-dihydro-4H-pyrano[3,2-c]quinoline-3-carbonitrile	[81]	200–400	-3.16	− 5.81*	Current dependence of light intensity, photoconductivity mechanism is controlled by monomolecular recombination in both diodes
2-amino-4-(2-Cl)-6-ethyl-5-oxo-5,6-dihydro-4H-pyrano[3,2-c]quinoline-3-carbonitrile	[81]	200–450	-3.16	− 6.11*	

HOMO and LUMO levels were determined either vs. vacuum (designated *) or vs. normal hydrogen electrode (NHE), designated **, as has been published in previous research

**Table 2:** Quinoline derivatives (metal complexes) and their properties

Chemical compound	References	Absorption bands [nm]	Energy levels		Others
			LUMO [eV]	HOMO [eV]	
tris-8-hydroxy-quino- linato aluminium (Alq3)	[82]	400–600	− 3.0	− 5.7*	
tris (8-hydroxyquinoline) aluminium	[83]	300–480	− 2.40	− 5.48*	Fluorescence lifetimes and relative amplitudes for the spin coated films of P3HT, Alq3 PMMA blends
[Ru(p-F-tpy)(pcqH)Cl]PF6 (F-TPY 40-(4-fluoro phenyl)-terpyridine, pcqH 2-(2-pyridyl)-4-carboxyquinoline)	[84]	250–700	− 2.98	− 5.74*	Thermogravimetry, TDDFT studies molecule optimization
2,6-bis(4-carboxy-5-chloroquinolin-2-yl)pyridine	[85]	200–900	0.38	− 4.11**	
[Ru{2,6-bis(4-carboxy-5-chloroquinolin-2-yl) pyridine}Cl_3_]	[85]	200–900	0.12	− 4.33	
2-bis[2-(2,2′-bithien-5-yl)-4- phenylquinolinato-C4,N]iridium(III) (2,4-pentanedionato-O2,O4) - [Ir(q-bt-Ph)2(acac)]	[86]	320–490	− 2.2	− 5.0*	Thermogravimetric analysis curves
4-carboxy-2-(2′-pyridyl) quinoline (Hmcpq)	[87]	250–900	0.74	− 0.91**	Infrared absorption spectra, adsorption studies (simple Langmuir)
cis-[Ru(H_2_dcpq)2(NCS)2] (2;H_2_dcpq′ 4-carboxy-2-[2′-(4′-carboxypyridyl)]quinoline)	[87]	250–900	0.48	− 0.74**	

HOMO and LUMO levels were determined either vs. vacuum (designated *) or vs. normal hydrogen electrode (NHE), designated **, as has been published in previous research

In Q1–Q4 materials, spacer remarkably affected the absorption spectra of the dyes. Materials Q1 and Q2 have a much weaker electron donor group (ethyl) than Q3 and Q4, which show clearly red-shifted bands. The lower HOMO level compared to those of Q1 and Q2 were connected with the presence of pyridocarbazole as a π-spacer in Q3 and Q4. It was obtained that the twisted molecular structure of Q4 results in HOMO levels is lower than for the Q3 material, whose structure was more linear. Q6 possesses the red-shifted and broadened absorption profile, which caused intensified charge separation and conjugation between donor and acceptor, then Q5. This improved electron-donating ability of the diphenylamine group. In Q5 was observed an efficient photoinduced electron transfer from the dye to the TiO_2_. The higher absolute value of LUMO level of Q6 is connected with the larger difference between the orbitals. Q7–10 have broad absorption spectra with two absorption bands (280–350 nm; 370–650 nm), which corresponds to the localized aromatic π–π* electron transition of the conjugated system. The changes in the absorption of Q8 and Q10 are due to two similar heteroaromatic rings. In the case of Q2, the heterocycle was quinazoline, while in Q4 it was replaced by a quinoline group. The difference between the two is the heterocycle lacking an electron attached to the acceptor unit. For the dyes with diphenylamine as donor (Q9) exhibits about red-shift in absorption relative to Q7 is observed a similar mechanism. As depicted in The HOMO orbital of these dyes is very similar, the LUMO orbitals show localized electron distribution through the cyanoacrylic acid and its adjacent p-spacer. Q11 and Q12 compounds have two absorption bands (200–400 nm and 200–450 nm, respectively), in which substitution of the phenoxyphenyl group shifts the absorption peaks towards high wavelength values. The energy levels were calculated using Anderson affinity rule [110].

Tris-8-hydroxy-quino-linato aluminium (Alq3), designated as Q13, is an organic electroluminescent material. With an absorption bandwidth of 300–600 nm, with one maximum around 350 nm. The authors presented slightly different values from different measurement methods. In the second case, the absorption spectrum presented was with 2-hydroxyethyl methacrylate. The Q14 shows two intense ligand-centred bands at 277 nm and 321 nm in the UV region. The o absorption at 277 nm is pcqH based, while the band at 321 nm is terpyridine based. Deprotonation of the –COOH group is connected with the large blue-shift of the peak position from 547 to 522 nm. The energy levels of Q16 are lower than Q15, especially, level. This reflects the connection with electron-withdrawing substituent, which was introduced to the 2,6-bis(4-carbox- yquinolin-2-yl)pyridine ligand. The absorption spectrum of Q17 includes the band from 320 to 490 nm, with a number of maxima (337 nm, 389 nm, 434 nm, 502 nm, 537 nm). The ruthenium complexes (Q18 and Q19) showed strong absorption bands in the region 250—360 nm, due to the π–π* transitions. Broad bands with 400 nm maximum (Q19) and 590 nm maximum for Q18 are also observed. Energy states of Q19 are more positive than Q18. It is linked to the introduction of a carboxyl group at the 4th position of H2dcpq, resulting in contribution to the stabilization of states.

## Applications

### PV cell

Pyrazoloquinoline-derived products can be applied in photovoltaic cells—in particular, organic photovoltaics, polymer photovoltaics, and dye-synthesized solar cells [107, 108]. Despite their low efficiency, these cells are among the fastest growing types of photovoltaic cells. The biggest advantage of polymer cells is the possibility of coating any surface with organic cells, which would place photovoltaics very close to people, and where they are needed as an energy source—worn on clothes, supporting tourism and sports, and generally being part of everyday life. DSSCs, on the other hand, are widely used in the construction industry due to their functional and design qualities [109, 110].

The crucial innovation that would allow organic photovoltaic systems to develop is the elimination of the classic p–n connector. Due to the polymeric character of these systems, charge transport (hopping process) is carried out between the donor and acceptor materials, precisely between their energy levels (HOMO and LUMO), in the whole volume of the active layer. The example donor:acceptor pair is P3HT:PCBM, which is made up of poly(3-hexylthiophene) and a fullerene derivative [6,6]-phenyl-C61-butyric acid methyl ester [111, 112].

The great advantage of quinoline derivatives is their agility and flexibility. They can be used both in polymer cells and DSSCs. In recent years, polymer solar cells and DSSCs based on the presented quinoline derivatives have been reported. The efficiency of photovoltaic devices is measured as power energy conversion or energy conversion efficiency (denoted as PQE, EQE or $\eta$) [113]. They provide the conversion of flux incident to device conversion into power. Sometimes Internal Quantum Efficiency (IQE)/Incident photon to convert electron (IPCE) is also reported—defined as the conversion of a photon to an electron at a given wavelength or charge carriers collected by a solar cell to a number of photons of a given energy [114].

The type of cells, architectures, and obtained efficiencies are detailed in Table 3. Cells with different electrodes, support layers, and, in DSSCs, different electrolytes have been reviewed. The performance of these cells ranges from 0.45% to over 4%. DSSCs based on quinoline and pyridocarbazole (Q1–Q2) solar cells efficiency increases in the order of Q1 (2.14%), Q2 (2.42%), Q3 (2.83%), and Q4 (2.92%), which is connected with broader absorption spectrum. In materials changing the donor group of methoxy in Q1 to three methoxy in Q2, they caused a slight increase in Voc.

**Table 3:** Solar cells based on quinoline derivatives (organic compounds): types and characteristics

Chemical compound	References	Cell type	Architecture	Compound name	Efficiency [%]
2-cyano-3-(2,6-dimethoxyquinolin-3-yl) acrylic acid	[78]	Dye solar cell	TiO_2_ coated FTO substrates	Electrolyte solution contains 2 mM dye and 0.1 M TBABF_4_ in DMSO, and the Fc/Fcþ	2.14
2-cyano-3-(2,5,6,7-tetramethoxyquinolin-3-yl) acrylic acid	[78]	Dye solar cell	TiO_2_ coated FTO substrates	Electrolyte solution contains 2 mM dye and 0.1 M TBABF_4_ in DMSO, and the Fc/Fcþ	2.42
2-cyano-3-(6-ethyl-2-methoxy-6H-pyrido[3,2-b]car- bazol-3-yl)acrylic acid	[78]	Dye solar cell	TiO_2_ coated FTO substrates	Electrolyte solution contains 2 mM dye and 0.1 M TBABF_4_ in DMSO, and the Fc/Fcþ	2.83
2-cyano-3-(7-ethyl-3-methoxy-7H-pyrido[2,3-c]carbazol-2-yl)acrylic acid	[78]	Dye solar cell	TiO_2_ coated FTO substrates	Electrolyte solution contains 2 mM dye and 0.1 M TBABF_4_ in DMSO, and the Fc/Fcþ	2.92
2-methyl-N,N-diphenylquinazolin-6-amine	[79]	Dye solar cell	TiO_2_-dye-electrolyte	Electrolyte: 0.6 M DMPImI þ 0.1 M LiI þ 0.1 M I2 in CH3CN solution;	0.72
N,N-bis(4-butoxyphenyl)-2-methylquinazolin-6-amine	[79]	Dye solar cell	TiO_2_-dye-electrolyte	Electrolyte: 0.6 M DMPImI þ 0.1 M LiI þ 0.1 M I2 in CH3CN solution; b Electrolyte: 0.6 M DMPImI þ 0.1 M LiI þ 0.1 M I_2_ þ 0.5 TPB in CH3CN	3.7
2-methyl-N,N-diphenylquinolin-6-amine	[80]	Dye solar cell	TiO_2_-dye-electrolyte	Electrolyte: 0.6 M DMPImI þ 0.1 M LiI þ 0.1 M I2 in CH3CN solution; b Electrolyte: 0.6 M DMPImI þ 0.1 M LiI þ 0.1 M I_2_ þ 0.5 TPB in CH3CN	1.77
N,N-bis(4-butoxyphenyl)-2-methylquinolin-6-amine	[80]	Dye solar cell	TiO_2_-dye-electrolyte	Electrolyte: 0.6 M DMPImI þ 0.1 M LiI þ 0.1 M I2 in CH3CN solution; b Electrolyte: 0.6 M DMPImI þ 0.1 M LiI þ 0.1 M I_2_ þ 0.5 TPB in CH3CN	2.51
(E)-2-cyano-3-(6-(diethylamino)quinolin-2-yl)acrylic acid	[80]	Dye solar cell	TiO_2_-dye-electrolyte	Electrolyte: 0.6MDMPImI + 0.1 M LiI + 0.05 M I_2_ + 0.5 TPB in CH3CN solution	0.72
(E)-2-cyano-3-(6-(3,6-dimethoxy-9H-carbazol-9-yl)quinolin-2-) acrylic acid	[80]	Dye solar cell	TiO_2_-dye-electrolyte	Electrolyte: 0.6MDMPImI + 0.1 M LiI + 0.05 M I_2_ + 0.5 TPB in CH3CN solution	0.75
2-Amino-6-ethyl-5-oxo-4-(3-Ph)-5, 6-dihydro-4H-pyrano[3,2-c]quinoline-3-carbonitrile	[81]	Polymer solar cell	Au/active layer/pSi/Al		1.2
2-Amino-4-(2-Cl)-6-ethyl-5-oxo-5,6-dihydro-4H-pyrano[3,2-c]quinoline-3-carbonitrile	[81]	Polymer solar cell	Au/active layer/pSi/Al		1.43

Q6 shows better coplanarity of the quinoline bridge with the pinwheel orientation of diphenylamine. It is connected with effective π-conjugation throughout the whole molecule skeleton, which facilitates the electron transfer between donor and acceptor materials. DSSC with Q6 (3.7%) material obtains the better results of charge recombination hindrance. It is connected with more efficient photoelectric conversion than in Q5 (0.72%)-based device.

The four-based quinoline (Q7-Q10) bearing butoxy groups- based dyes sensitized solar cells obtained a short circuit photocurrent density of 7.04 mA/cm^2^ and open circuit voltage of 0.52 V. The overall conversion efficiency of 2.51% was obtained. The highest efficiency of the Q10-based cell is due to the increase in the short current circuit, caused by a broader absorption spectrum as well as a higher molar extinction coefficient. The LUMO energy levels are connected with the electron injection capability, which directly affects the short current circuit. The Voc of the Q10-based cell was higher than that of Q8-based cells, which was found that quinoline p-spacer also improves the Voc and the effects on efficiency. The cells based on Q11 and Q12 were prepared and manufactured. The performances of the devices were 1.43% and 1.26%, respectively.

Alq3 material (Q13) was used as a dopant in the buffer layer (electron-transporting layer), as an addition to bathocuproine. It has an absorption with a broad bandwidth of 300–600, with a single maximum around 350 nm. This also increased the energy difference between the LUMO of the acceptor layer and the HOMO of the donor layer, which resulted in an increase in the open-circuit voltage. Doping Q13 increased photocurrent explained by the broader spectral retraction. Q13 was also used as an additive ingredient to a typical organic active layer consisting of P3HT and PCBM with 2.24% efficiency. Photovoltaic measurements with the DSSC device sensitized with Q14 dye were performed. The quantum efficiency was obtained equal to 13%, with open-circuit photovoltage 0.35 V and short-circuit current density 1.428 A/cm^2^. For the Q16-based DSSC, devices afforded higher efficiency (3.0* and 2.7**, depending on the design) than for Q15 cells (1.7* and 0.5**, depending on the design). It should also be mentioned that Q15 showed 35% incident high photon-to-current efficiency (IPCE) at 900 nm, which definitely has an impact on performance. The absorbance is also higher than for Q16, especially in the near-IR region. Adding to a donor (P3HT) and acceptor (PCBM) as the third component of Q17 resulted in performance below that of typical ITO/PEDOT:PSS/active layer/aluminium architecture (0.25%). Photovoltaic solar cells based on TiO_2_ sensitized with Q18 and Q19 showed 1.2% and 1.45% efficiency, respectively. The short-circuit current density is related to the incident IPCE. They are approximate and showed 50% for the 600 nm wavelength. The efficiency of the Q18-based sensitized solar cells was reduced in relation to Q19, which corresponds to the excess coverage of the molecules on the TiO_2_ (Table 4).

**Table 4:** Solar cells based on quinoline derivatives (metal complexes): types and characteristics

Chemical compound	References	Cell type	Architecture	Compound name	Efficiency [%]
Tris-8-hydroxy-quino- linato aluminium (Alq3)	[82]	Polymer solar cell	ITO/CuPc/active layer/buffer layer/Al		1.76
Tris (8-hydroxyquinoline) aluminium	[83]	Polymer solar cell	ITO/ZnO/active layer/buffer/Au		2.24
[Ru(p-F-tpy)(pcqH)Cl]PF6 (F-TPY 40-(4-fluoro phenyl)- terpyridine, pcqH 2- (2-pyridyl)-4-carboxyquinoline)	[84]	Dye solar cell	TiO_2_ electrodes	electrolyte, composed of 0.5 M NaI, 0.05 M I_2_ 0.05 M Triphenylmethylphosphonium iodide	0.13
2,6-Bis(4-carboxy-5-chloroquinolin-2-yl)pyridine	[85]	Dye solar cell		LiI-rich electrolyte made of 2 M LiI and 0.05 M I_2_ in acetonitrile, *LiI/M: 2 **LiI/M:0.1DMPmI/M 0.6	3.0* 2.7**
[Ru{2,6-bis(4-carboxy-5-chloroquinolin-2-yl) pyridine}Cl_3_]	[85]	Dye solar cell		LiI-rich electrolyte made of 2 M LiI and 0.05 M I_2_ in acetonitrile, *LiI/M: 2 **LiI/M:0.1DMPmI/M 0.6	1.7* 0.5**
2—Bis[2-(2,2′-bithien-5-yl)-4- phenylquinolinato-C4,N]iridium(III) (2,4-pentanedionato-O2,O4) - [Ir(q-bt-Ph)2(acac)]	[86]	Polymer solar cell	ITO/PEDOT:PSS/P3HT:PCBM:QDD/Al		0.25
4-carboxy-2-(2′-pyridyl) quinoline (Hmcpq)	[87]	Dye solar cell	TiO_2_-dye-electrolyte	The electrolyte solution was composed of 0.6 M 1,2-dimethyl-3-propylimidazolium iodide, 0.05 M I_2_, and 0.1 M LiI in acetonitrile	4.6
cis-[Ru(H_2_dcpq)2(NCS)2] (2;H_2_dcpq′ 4-carboxy-2-[2′-(4′-carboxypyridyl)] quinoline)	[87]	Dye solar cell	TiO_2_-dye-electrolyte	The electrolyte solution was composed of 0.6 M 1,2-dimethyl-3-propylimidazolium iodide, 0.05 M I_2_, and 0.1 M LiI in acetonitrile	4.3

HOMO and LUMO levels were determined either vs. vacuum (designated *) or vs. normal hydrogen electrode (NHE), designated **, as has been published in previous research

### Other applications

A photovoltaic cell is a semiconductor element through which the energy of solar radiation is converted into electricity as a result of a photovoltaic phenomenon. The current–voltage characteristic of a solar cell (organic and dye) is the current–voltage characteristic of a diode; the electrical current is conducted asymmetrically. Therefore, solar cells are diodes with many features.

A different subgroup of diodes are electroluminescent diodes. For quinoline derivatives, these are organic light-emitting diodes (OLEDs). Tang [115, 116] displays based on OLED technology are becoming increasingly popular. Their advantages are highly saturated colours, low energy consumption, and the ability to be produced as large, flexible surfaces. The mechanism of light emission from an organic material involves injects a charge from an electrode into the material, which must be accomplished by crossing or tunnelling through the barrier of the metal–polymer junction. The properties required of the materials for application in diodes are similar to those required for photovoltaic solar cells. Some materials can be successfully used to produce both solar cells [117] and OLEDs [118]. Chemical compounds of pyrazoloquinoline derivatives have also been successfully used in OLED emission layers [119, 120].

Other important electrical elements are organic transistors. Organic field transistors (OFETs) are designed to detect gaseous and liquid compounds. OFET purposes, substrate type, and manufacturing processes, as well as the physical properties of the materials used to make OFETs, may dictate the choice of appropriate layered structures. Currently, OFET parameters are similar to those produced on amorphous silicon, and their commercialization is slowed down only by the low operational stability and resistance to external factors [121]. Quinoline derivatives have been used in organic transistors. The heptacyclic bisindoloquinoline-based materials were used to fabricate single-crystal field-effect transistors [122]. Static induction transistors with various organic semiconductor materials, such as mentioned as OLED material tris(quinoline-8-hydroxylate) aluminium [123], were also fabricated and studied. Organic field-effect transistor (OFET) devices were fabricated using ester-flanked quinolones [124].

Quinoline derivatives are best known for their medical applications [125]. They are pretested as inhibitors for treating diabetes [126]; they are also used as antiviral and cancer drugs [126] and others [127]. Most famously, quinine was the first effective antimalarial drug [128, 129]. Derivatives obtained by hybridization indicate anti-cancer applications [130, 131]. The popular group of drugs based on a quinoline fragment is the quinolones. More than 10 000 have already been described; several have been registered as drugs [132]. Alq8 mentioned in photovoltaic applications is also a promising antibiotic against *Staphylococcus aureus* [133]. Recently, derivatives have been typed for COVID-19 [134]. The area of medical applications for these derivatives is so large that it requires a separate review.

## Conclusions

The most recent knowledge on the selected quinoline derivatives, which have been successfully used to produce photovoltaic cells, were reviewed. Furthermore properties were discussed relevant to the application of these derivatives, and the reference data and information were collected for a selection of compounds. The properties of the obtained quinoline derivatives are diversified, depending on the chosen substituents. The optoelectronic properties can be controlled by the structure of the molecule, which is a definite advantage. Both HOMO and LUMO levels as well as absorption spectra occur in different values and ranges, related to the chemical structure of quinoline derivatives. The remaining cell parameters are also varied due to different manufacturing techniques and architectures. These diverse achievements indicate that the optimization and testing of quinoline-based cells pose an interesting problem, and further research is needed. PV cells and quinoline-based devices in general are improved and are still a topical issue [107, 135, 136]. The photovoltaic cells made so far in different configurations and architectures have shown limited efficiency (up to 5%), but it should be remembered that this subject is still under development. Many other applications, either electronic (OLEDs, transistors) or medical, should also be appreciated. Despite actually lower than average efficiency, due to the above features and advantages, quinolines are an important part of chemical materials research.
